# Supplementary material for: Development of an adenosquamous carcinoma histopathology – selective lung metastasis model
Source: Biol Open. 2022 Dec 13;11(12):bio059623. doi: 10.1242/bio.059623 (PMC9770245; doi:10.1242/bio.059623)
Supplement: Supplementary information [file biolopen-11-059623-s1.pdf]

Biology Open (2022): doi:10.1242/bio.059623: Supplementary information

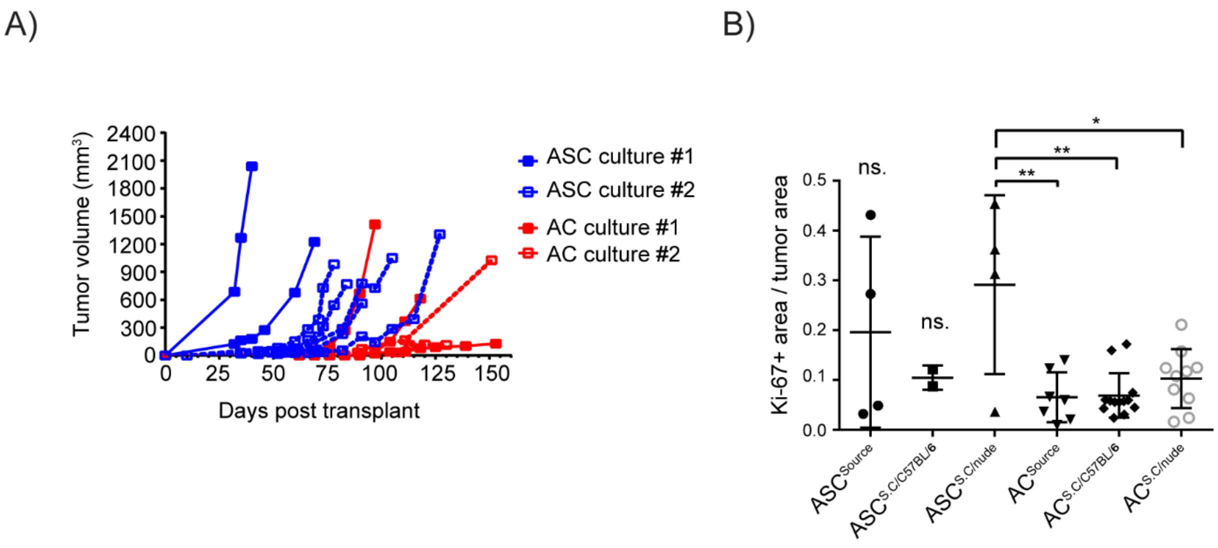

Fig. S1. Host microenvironment-dependent and histopathology-selective tumor establishment subcutaneously. (A) Subcutaneous ASC or AC tumor growth curves in athymic nude recipient mice using *in vitro* cultured cells. Each line represents one mouse and the tumors were measured weekly by using a caliper. n=7 (ASC), 5 (AC). **(B)** ASC and AC cells were subcutaneously transplanted and post-mortal IHC analysis of Ki-67 was performed (Figure 1 C-D). Quantification of Ki-67 positive area by dividing the positive tumor area with the total tumor area in each tumor separately by using FIJI ImageJ. The graph represents mean+/- SD for each group, and each point represents an individual tumor. Two-tailed unpaired Student's t-test values are *p<0.05, **p<0.01. n=3 (2 for ASC$^{s.c.C57BL/6}$).

Biology Open • Supplementary information

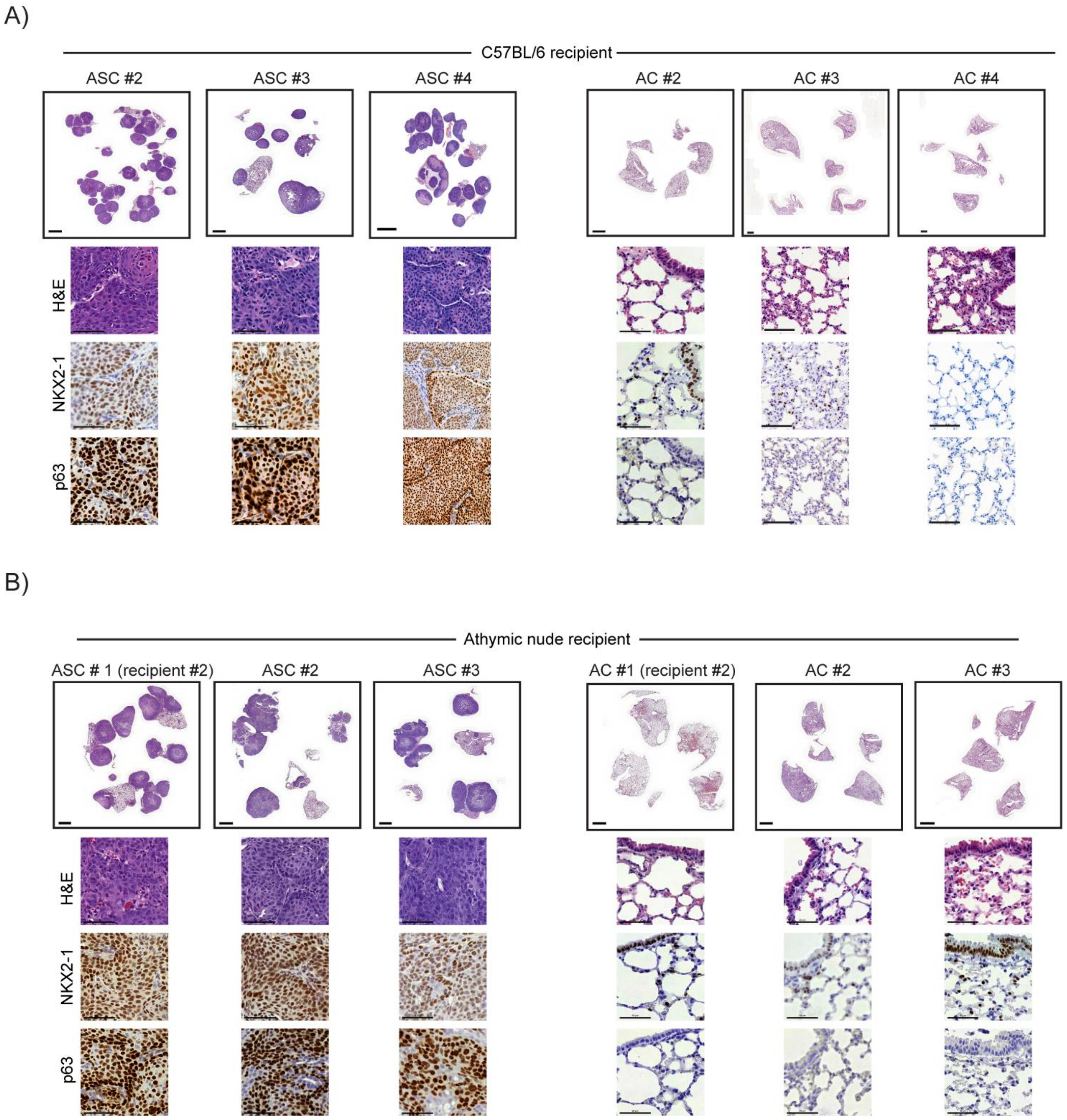

Fig. S2. Host microenvironment-dependent and histopathology-selective tumor establishment intravenously. (A-B) Biological replicates for Figure 2 with H&E, nkx2-1 and p63, IHC images of C57BL/6 recipient or athymic nude mice upon i.v. transplantation with ASC or AC tumor-derived cells. Scale bar 2 mm (2x) or 50 µm (40x). n=6.

Biology Open • Supplementary information

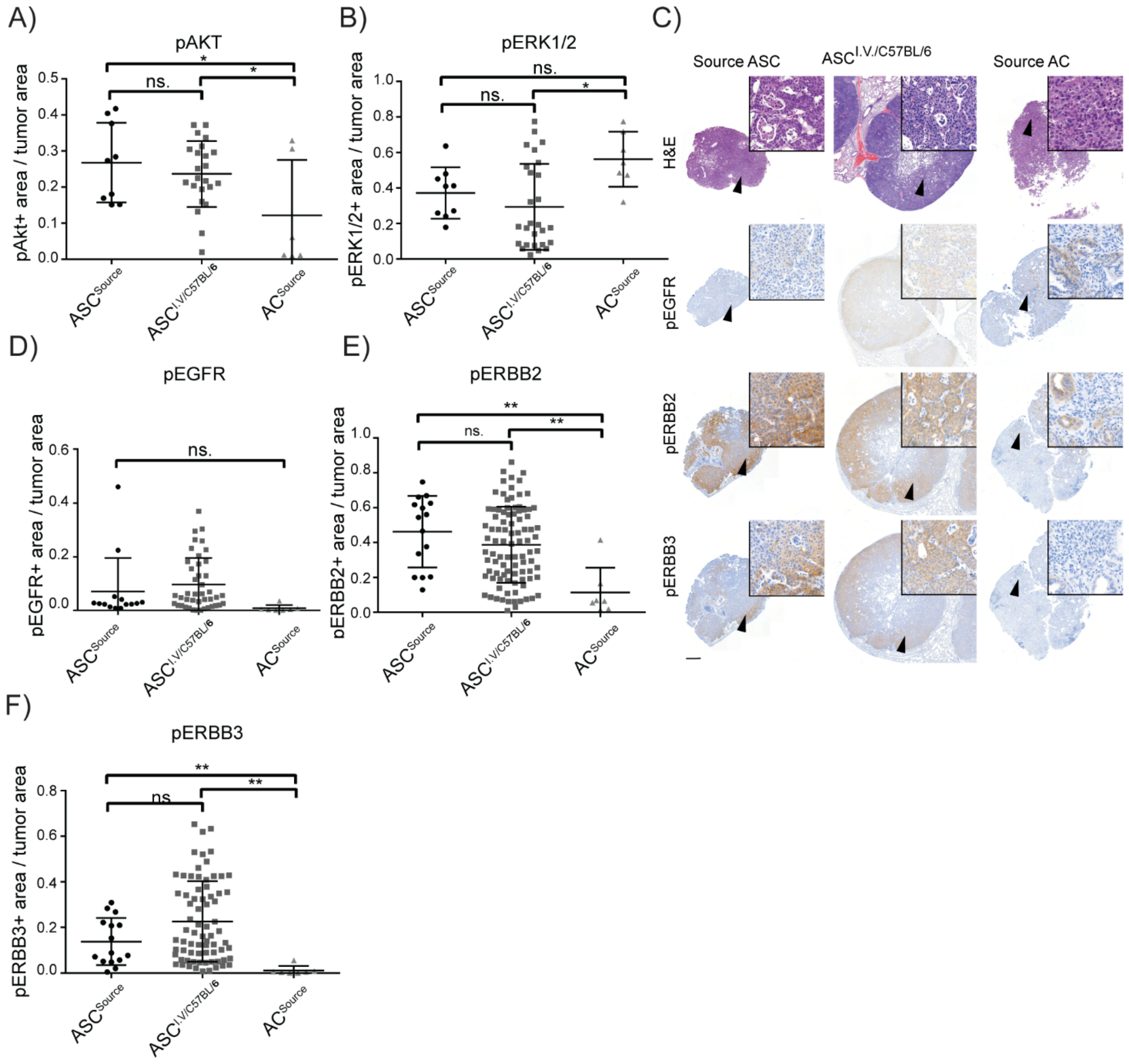

Fig. S3. ASC$^{i.v.}$ cells mimic KL;ASC tumors in showing increased pERBB levels compared to KL;AC mice. (A-B) Quantification of pAKT and pERK positive area by dividing the positive tumor area with the total tumor area in each tumor separately by using FIJI ImageJ. The graph represents mean+/- SD for each group, and each point represents an individual tumor. Two-tailed unpaired Student's t-test values are *p<0.05. n=5. **(C)** Representative H&E, pEGFR, pERBB2 and pERBB3 IHC images of KL;ASC source, i.v. tumors from C57BL/6 recipient mice upon transplantation with ASC tumor-derived cells and KL;AC source tumors. Scale bar 500 μm (2x) or 20 μm (40x). n=5 **(D-F)** Quantification of pEGFR, pERBB2 and pERBB3 positive area by dividing the positive tumor area with the total tumor area in each tumor separately by using FIJI ImageJ. The graph represents mean +/- SD for each group, and each point represents an individual tumor. Two-tailed unpaired Student's t-test values are *p<0.05, **p<0.01. n=5.

Biology Open • Supplementary information

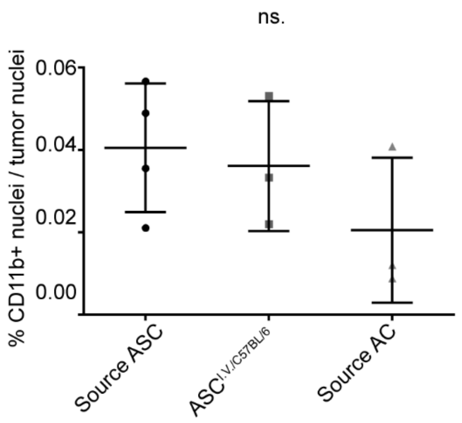

Fig. S4. No differences are measured in the number of CD11b+ neutrophils in KL;ASC source and ASC$^{i.v.C57BL/6}$ tumors. Quantification of CD11b positive nuclei by dividing the positive tumor nuclei with the total tumor nuclei in each tumor separately by using FIJI ImageJ and CellProfiler. The graph represents mean+/- SD for each group, and each point represents an individual mouse. n=4.

Biology Open • Supplementary information

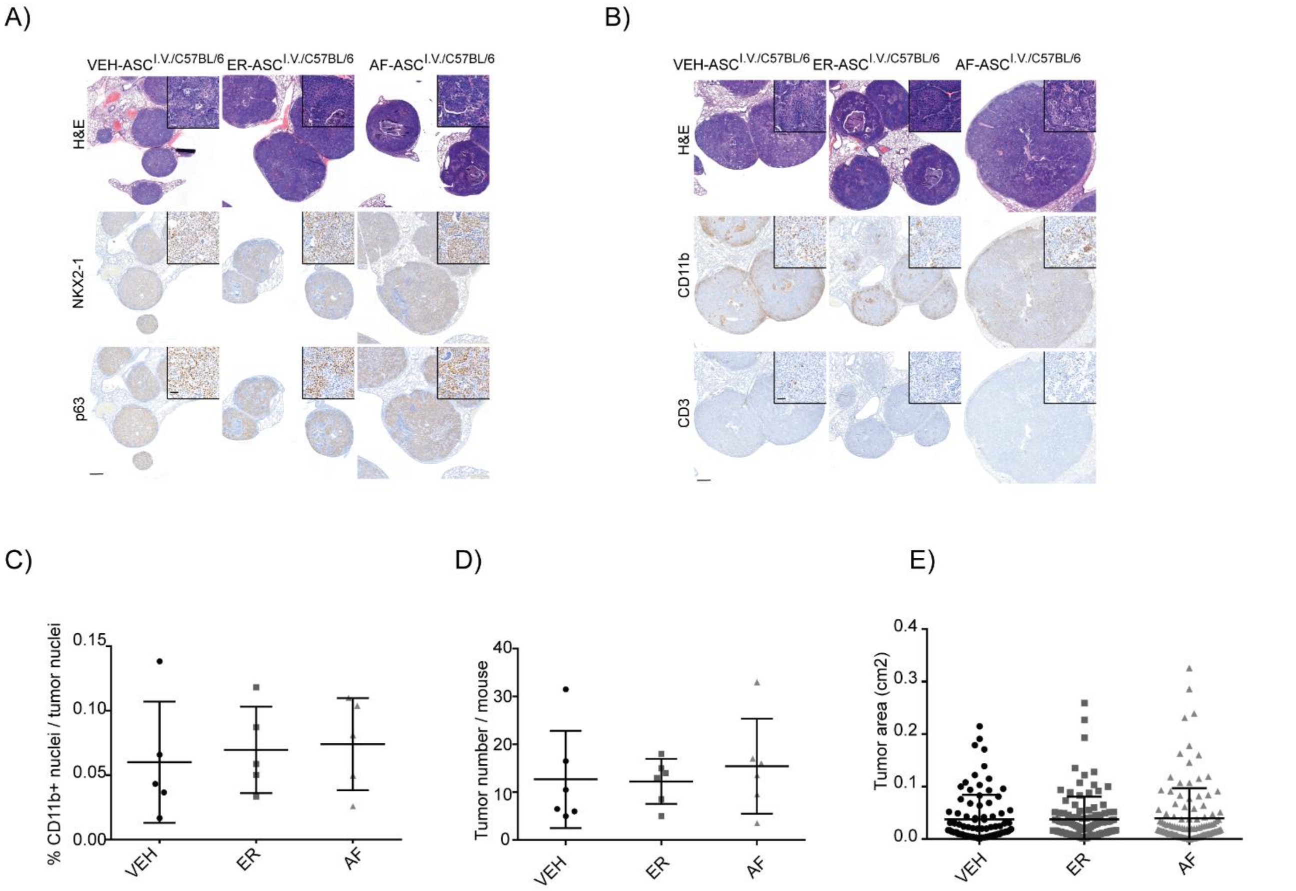

Fig. S5. Therapeutic treatment of ASC$^{i.v.}$ mice does not associate with differences in tumor histotype, immunophenotype or tumor size. (A) ASC$^{i.v.}$ mice were treated 10 days after intravenous transplantation with vehicle (VEH), erlotinib (ER) or afatinib (AF). Representative H&E, nkx2-1 and p63 IHC images of VEH, ER and AF treated mice. Scale bar 500 µm (2x) or 20 µm (40x). n=5. **(B)** Representative H&E, CD11b and CD3 IHC images of VEH, ER and AF treated mice. Scale bar 500 µm (2x) or 20 µm (40x). n=4-5 **(C)** Quantification of CD11b positive nuclei by dividing the positive tumor nuclei with the total tumor nuclei in each tumor separately by using FIJI ImageJ and CellProfiler. The graph represents mean +/- SD for each group, and each point represents an individual tumor. n=4. **(D)** The tumor number after VEH, ER or AF treatment was derived from the H&E stainings. The graph represents mean +/- SD for each group, and each point represents an individual mouse. n=6. **(E)** The tumor area after VEH, ER or AF treatment was derived from the H&E stainings using Fiji ImageJ. The graph represents mean +/- SD for each group, and each point represents an individual tumor. n=6.

Biology Open • Supplementary information

Table S1. List of antibodies and their concentrations used for FACS and IHC analysis.

Antibody	Company	Code	Contentration	Notes - antigen retrieval
p63	Abcam	ab124762	1:10000	Citric acid pH 6
pAKT	Cell Signaling Tech.	CST4058	1:400	Citric acid pH 6
CD11b	Bio SB	BSB6441	1:250	Citric acid pH 6
CD11b-FITC	Biolegend	101206	1:100	for FACS
CD3	Abcam	ab5690	1:1000	Citric acid pH 6
CD45- PE	E-Biosciences	12-0451-82	1:200	for FACS
pERK1/2	Cell Signaling Tech.	CST4370	1:1000	Citric acid pH 6
pEGFR	Cell Signaling Tech.	CST2234	1:50	Tris-EDTA pH 9
pERBB2	Cell Signaling Tech.	CST2243	1:200	Tris-EDTA pH 9
pERBB3	Cell Signaling Tech.	CST4791	1:200	Tris-EDTA pH 9
Gr1- APC	Biolegend	108412	1:200	for FACS
KI67	Thermofisher Scientific	MA-14520	1:200	Tris-EDTA pH 9
NKX2-1	Abcam	ab133638	1:2000	Citric acid pH 6
Vimentin	Cell Signaling Tech.	CST5741	1:500	Tris-EDTA pH 9

Biology Open • Supplementary information
